# The Results of 16 Years Iodization: Assessment of Iodine Deficiency Among School-age Children in Antalya, Turkey

**DOI:** 10.4274/jcrpe.galenos.2019.2019.0036

**Published:** 2020-09-02

**Authors:** Zheng Feei Ma

**Affiliations:** 1 Department of Health and Environmental Sciences, Xi’an Jiaotong-Liverpool University, Suzhou, China; School of Medical Sciences, Universiti Sains Malaysia, Kota Bharu, Kelantan, Malaysia

**Keywords:** Iodine, urinary iodine concentration, children, Turkey

## Dear Editor,

This letter is regarding the recent publication of “The results of 16 years iodization: Assessment of iodine deficiency among school-age children in Antalya, Turkey” by Çelmeli et al (1) (2020). The study consisted of 1594 children aged 6-14 years who were asked to provide spot urine samples for the determination of urinary iodine concentration (UIC) after informed consent form was obtained from the parents of the children (1). In addition, goiter rate was also assessed in the study (1).

Iodine deficiency is one of the most common micronutrient deficiencies which can be prevented with the implementation of universal salt iodization programme (2). However, like any other health intervention programmes, the monitoring and evaluation of universal salt iodization programmes are needed to conducted regularly. This is particularly important to ensure that timely information is obtained so that a population will not be getting too much or too little iodine from the universal salt iodization programmes. Also, this would allow for an adjustment of iodized salt concentration to meet the recommended iodine intake in populations (3). In the study, the authors reported a median UIC of 175 µg/L with 19% of children categorised as mild-to-moderate iodine deficiency. Mild-to-moderate iodine deficiency has been shown to affect the learning ability and cognition of children (2). Therefore, if these children remain to be iodine deficient without appropriate corrective measures taken, these children are mostly likely to have a lower cognition and intelligence quotient (2).

Median UIC is the recommended method by World Health Organization, UNICEF (United Nations Children’s Fund) and IGN (Iodine Global Network) to assess iodine status in populations (2). Urinary iodine in spot urine samples can also be expressed as iodine-to-creatinine ratio (I/Cr) ratio and estimated 24-hour urine iodine excretion (2). Although the collection of spot urine samples for the determination of UIC is relatively easier to be performed, UIC has high intra- and inter-individual variation (2). In addition, UIC is also subjected to hydration status (2). Therefore, Cr has been proposed to be included in the assessment of UIC in order to minimize the effect of hydration status (4). However, relating UIC to Cr might be unnecessary because this adjustment might introduce another bias from Cr and increase the cost of assessing iodine status using UIC. This is because when a population is malnourished or having low protein intake, the excretion of Cr is usually low and this can confound I/Cr ratio. There are no acceptable criteria for determining iodine sufficiency using I/Cr ratio. In addition, the use of iodized salt in populations should be reported. A low coverage of iodized salt might put populations at risk of iodine deficiency in future (3).

## References

[1] Çelmeli G, Çürek Y, Özen Küçükçetin İ, Arslan Gülten Z, Özdem S, Akçurin S, Bircan İ (2020.). The results of 16 years iodization: assessment of iodine deficiency among school-age children in Antalya, Turkey. J Clin Res Pediatr Endocrinol.

[2] Zhou H, Lu Y, Pan B, Ma ZF. Iodine deficiency as assessed by neonatal TSH in a sample of mother-and-newborn pairs in Jiangsu Province, China. Biol Trace Elem Res 2020. Available from: https://doi.org/10.1007/s12011-020-02135-6.

[3] Ma ZF (2019). A comparative study of iodised salt programs: Shanghai and Switzerland. Biol Trace Elem Res.

[4] Ma ZF, Venn BJ, Manning PJ, Cameron CM, Skeaff SA (2018). The sensitivity and specificity of thyroglobulin concentration using repeated measures of urinary iodine excretion. Eur J Nutr.

